# Bidirectional two-sample Mendelian randomization analyses support causal relationships between structural and diffusion imaging-derived phenotypes and the risk of major neurodegenerative diseases

**DOI:** 10.1038/s41398-024-02939-3

**Published:** 2024-05-28

**Authors:** Zirui Wang, Xuan Yang, Haonan Li, Siqi Wang, Zhixuan Liu, Yaoyi Wang, Xingyu Zhang, Yayuan Chen, Qiang Xu, Jiayuan Xu, Zengguang Wang, Junping Wang

**Affiliations:** 1 Department of Radiology, Tianjin Key Lab of Functional Imaging & Tianjin Institute of Radiology, Tianjin Medical University General Hospital, Tianjin, 300052 China; 2 Department of Radiology, Jining No.1 People’s Hospital, Jining, Shandong 272000 China; 3 Department of Neurosurgery, Tianjin Medical University General Hospital, Tianjin, 300052 China

**Keywords:** Molecular neuroscience, Clinical genetics, Diagnostic markers

## Abstract

Previous observational investigations suggest that structural and diffusion imaging-derived phenotypes (IDPs) are associated with major neurodegenerative diseases; however, whether these associations are causal remains largely uncertain. Herein we conducted bidirectional two-sample Mendelian randomization analyses to infer the causal relationships between structural and diffusion IDPs and major neurodegenerative diseases using common genetic variants-single nucleotide polymorphism (SNPs) as instrumental variables. Summary statistics of genome-wide association study (GWAS) for structural and diffusion IDPs were obtained from 33,224 individuals in the UK Biobank cohort. Summary statistics of GWAS for seven major neurodegenerative diseases were obtained from the largest GWAS for each disease to date. The forward MR analyses identified significant or suggestively statistical causal effects of genetically predicted three structural IDPs on Alzheimer’s disease (AD), frontotemporal dementia (FTD), and multiple sclerosis. For example, the reduction in the surface area of the left superior temporal gyrus was associated with a higher risk of AD. The reverse MR analyses identified significantly or suggestively statistical causal effects of genetically predicted AD, Lewy body dementia (LBD), and FTD on nine structural and diffusion IDPs. For example, LBD was associated with increased mean diffusivity in the right superior longitudinal fasciculus and AD was associated with decreased gray matter volume in the right ventral striatum. Our findings might contribute to shedding light on the prediction and therapeutic intervention for the major neurodegenerative diseases at the neuroimaging level.

## Introduction

Neurodegenerative diseases encompass a collection of chronic and progressive disorders characterized by the gradual degeneration and loss of neurons in specific areas of the central nervous system which impose substantial public health and economic burdens throughout the world and are nowadays the major and increasing causes of disability and death [1, 2]. Major neurodegenerative diseases include Alzheimer’s disease (AD), Parkinson’s disease (PD), amyotrophic lateral sclerosis (ALS), multiple sclerosis (MS), Lewy body dementia (LBD), frontotemporal dementia (FTD), and Huntington’s disease (HD). AD causes progressive decline in cognitive abilities including memory impairment, confusion, alterations in personality, and a gradual loss of autonomy and self-sufficiency, and is the most prevalent cause of dementia among elderly individuals [3]. PD is distinguished by asymmetrical bradykinesia, muscular stiffness, resting tremors, compromised balance, impaired speech and swallowing abilities, and disturbances in autonomic reflexes [4]. ALS manifests as a progressive loss of motor neurons, which mainly involves corticopontine and corticospinal tracts [5]. MS is a chronic inflammatory condition characterized by the pathological hallmark of massive leukocyte infiltration and extensive microglial activation, which results in neuroaxonal demyelination and degeneration. LBD is the second most prevalent form of dementia, which manifest as a tetrad of cognitive impairment, visual hallucinations, spontaneous parkinsonism, and rapid eye movement sleep behaviour [6]. FTD is the third most prevalent form of dementia following AD and LBD and is a leading type of early-onset dementia which is an insidious clinical syndrome characterized by progressive deficits in behaviour, executive control, language, and motor symptoms [7]. HD is an autosomal dominant disorder leading to a progression of involuntary choreiform movements, psychological change, and dementia [8].

A large number of case-control investigations have revealed that individuals diagnosed with major neurodegenerative diseases have structural changes in a wide range of brain regions, including many heritable imaging-derived phenotypes (IDPs) that can be measured by non-invasive magnetic resonance imaging (MRI) technique. For instance, atrophy of the entorhinal cortex and hippocampus is a key neuropathological feature of AD patients, however, the atrophy of the hippocampus could also be observed in patients with LBD, PD, and HD, although the atrophy level was less than that in patients with AD [9–12]. Investigations have also identified atrophy of the cingulate cortex in multiple neurodegenerative diseases, such as AD, MS, and FTD [9, 13, 14]. Fractional anisotropy (FA) is a diffusional measure that can reflect the white matter integrity, and mean diffusivity (MD) provides a measure of the average extent of molecular displacement resulting from diffusion processes. Reduced FA and increased MD are commonly interpreted as indicators of disrupted fibre tracts and demyelination. The changes in these two measures are highly sensitive in detecting early deviations in the microstructural integrity of white matter and are linked to a range of neuropathological alterations [15]. Patients with AD and LBD both demonstrated reduced FA and increased MD in various brain regions, including the temporal, parieto-occipital, and frontal lobes [16]. In addition, individuals diagnosed with ALS experienced a gradual decline in FA of the corticospinal tract and frontal lobes [17]. However, the role of the abovementioned structural and diffusion IDPs alterations in each disease may differ. Some may be the aetiology of the disease, while others may reflect the pathological and physiological changes during the course of the disease. Moreover, the results obtained from conventional observational studies might be less reliable due to confounding factors such as medications or environment/lifestyle changes and reserve causation.

**Fig. 1:**
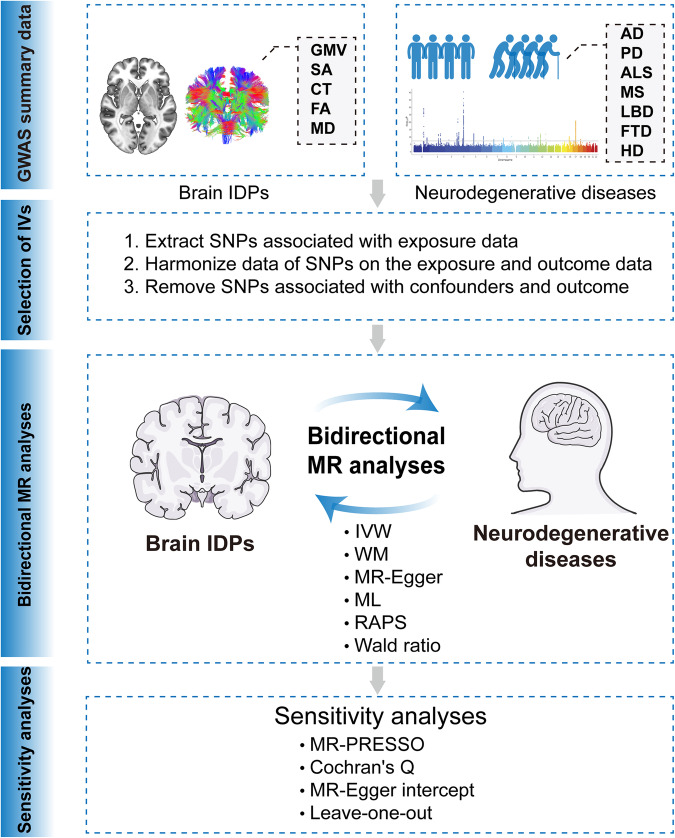
Study flowchart of the causal relationships between structural and diffusion IDPs and the risk of major neurodegenerative diseases by using bidirectional TSMR analysis methods. AD Alzheimer’s disease, ALS amyotrophic lateral sclerosis, CT cortical thickness, FA fractional anisotropy, FTD frontotemporal dementia, GMV gray matter volume, HD Huntington’s disease, IDPs imaging-derived phenotypes, IVW inverse-variance weighted, LBD Lewy body dementia, MD mean diffusivity, ML maximum likelihood, MS multiple sclerosis, PD Parkinson’s disease, RAPS MR-robust adjusted profile scores, SA surface area, TSMR two-sample Mendelian randomization, WM weighted median.

Mendelian randomization (MR) is a novel approach to make causal inference between an exposure (or a risk factor) and an outcome by using genetic variants associated with the exposure as instrumental variables (IVs) (e.g., single nucleotide polymorphisms [SNPs]) [18]. Due to the random assortment of alleles at conception, the distribution of genetic variants that are associated with a particular exposure is largely independent of the factors that confound exposure-outcome associations in conventional observational studies [19]. Therefore, the estimates from MR analyses are less affected by the confounding factors, and can provide more reliable insight into the causal relationships between the exposure and the outcome than classical observational studies. In addition, given that the alleles of an individual are determined at conception and cannot be modified by subsequent outcomes, the direction of causation is always from the genetic variants to the outcomes, and therefore, it eliminates the potential bias due to reverse causation. Since an individual’s alleles are determined at conception and random assortment, the MR approach avoids some of the classical observational study limitations and provides more robust causal evidence, which is unaffected by confounding factors and avoids potential bias arising from reverse causation. When performing MR analysis, three assumptions must be satisfied: 1) the IVs are strongly associated with the exposure; 2) the IVs are independent of any potential factors that confound the association of the exposure with the outcome; and 3) the IVs affect the outcome only through the exposure [18]. With the increase of the publicly available summary statistics of genome-wide association studies (GWAS) on brain IDPs [20–22] and neurodegenerative diseases [23, 24], using the MR analysis method to infer the causal relationship between them has become feasible. To our knowledge, there are four studies investigating the causal associations between brain IDPs and AD using the MR analysis approach, with the sample size of IDPs ranging from 17,467 to 33,992 and the sample size of AD (or AD-by-proxy) ranging from 21,982 to 71,880 [25–28]. The IDPs selected in these studies are mainly structural and diffusion metrics of cortex and white matter tracts, with the number ranging from 3 to 221. Only two studies revealed suggestive causal associations between IDPs and AD. For example, Wu et al. [26] carried out a bidirectional two-sample MR (TSMR) analyses to infer the causal associations between cortical metrics (surface area [SA] and cortical thickness [CT] of the entire cortex and 34 brain regions, 33,992 participants) and the risk of AD (71,880 AD [or AD-by-proxy]; 383,378 controls). They found suggestive causal associations of atrophy of the temporal pole and cuneus with increased risk of AD and vulnerability to AD with decreased SA of the precentral and isthmus cingulate. Song et al. [25] conducted TSMR analyses to infer the causal effects of 110 diffusion tensor imaging (DTI) IDPs (FA, MD, axial diffusivity, mode of anisotropy, and radial diffusivity; 17,706 participants) and 101 structural IDPs (gray matter volume [GMV], 19,629 participants) on the risk of AD (71,880 AD [or AD-by-proxy]; 383,378 controls) and 10 psychiatric disorders and found a suggestive causal link between decreased FA of corpus callosum body and increased AD risk. These studies merely focused on the causal relationship between AD (single neurodegenerative disease) and brain IDPs. Moreover, Wu et al.’s [26] study only included a limited number of IDPs; and Song et al.’s [25] study was based on a relatively small sample size of GWASs for IDPs. In the current study, we conducted bidirectional TSMR analyses to systematically assess the causal relationships between structural and diffusion IDPs and seven major neurodegenerative diseases (AD, PD, ALS, MS, LBD, FTD, and HD). Our findings might contribute to elucidating the etiology of these major neurodegenerative diseases at the neuroimaging level and brain structural alterations in the course of neurodegenerative diseases and thus guide early-stage prevention and treatment. An overview of our study design is summarized in Fig. 1.

## Materials and methods

### Structural and diffusion IDPs

Smith et al. [24] conducted a GWAS using 3913 brain IDPs in 33,224 participants of European descent from the 2020 release of UK Biobank (UKBB), and all GWAS summary statistics can be downloaded from the Oxford Brain Imaging Genetics (BIG40) web browser (https://open.win.ox.ac.uk/ukbiobank/big40/). According to the findings of Smith et al. [23], the heritabilities of structural and diffusion IDPs are significantly higher than those of functional IDPs; for DTI, the heritabilities of the tract-based spatial statistics (TBSS) IDPs are higher than those of the tractography-based IDPs. Therefore, we selected the structural (T1-weighted image) and TBSS-based DTI IDPs for the following MR analyses [29]. For the structural IDPs, we chose the most commonly used metrics: GMV (unit: mm^3^), surface area (SA, unit: mm^2^), and cortical thickness (CT, unit: mm).

One hundred and nine GMV IDPs including 66 cortical, 15 subcortical, and 28 cerebellar regions were chosen based on Desikan-Killiany cortical, Harvard-Oxford subcortical, and Diedrichsen cerebellar atlases, respectively. Sixty-eight SA and 68 CT IDPs were chosen based on the Desikan-Killiany atlas. For the diffusion IDPs, we selected the most commonly used measures (FA and MD [unit: mm^2^/s]) which could reflect the integrity of the white matter microstructure. Forty-eight FA and 48 MD IDPs were selected by using the Johns Hopkins University DTI Atlas. Finally, a total of 341 structural and diffusion IDPs were chosen in our study. Detailed information on our selected 341 IDPs is provided in Supplementary Table 1.

### Neurodegenerative diseases

Seven major neurodegenerative diseases with publicly available GWAS summary statistics were collected in our study, including AD (21,982 cases, 41,944 controls) [20], PD (33,674 cases, 449,056 controls) [21], ALS (27,205 cases, 110,881 controls) [30], MS (47,429 cases, 68,374 controls) [31], LBD (2591 cases, 4027 controls) [32], FTD (2154 cases, 4308 controls) [22], and HD (9064 cases) [33]. All the summary statistics included in our study were from the largest GWASs to date for the onset risk of neurodegenerative diseases, except the GWAS for AD and HD. For AD, we selected the GWAS summary statistics from the International Genomics of Alzheimer’s Project (IGAP), which comprised 21,982 late-onset AD patients and 41,944 cognitively normal controls [20]. Although there have been more recent AD GWASs published in 2021 [34] and 2022 [35] with larger sample sizes, more than half of the cases in these two investigations are AD-by-proxy from the UKBB. The UKBB’s AD-by-proxy designation is based on the question of whether the individuals’ parent had dementia (all types of dementia, not just AD-related dementia). This relatively less specific method increases heterogeneity in survey data and therefore leads to systematic biases [36]. For instance, a recent preprint paper revealed that current AD GWAS-by-proxy practices provide highly misleading genetic correlations between AD risk and higher education [37]. Therefore, we opted for the IGAP dataset to avoid biases associated with GWAS-by-proxy. Additionally, considering that the brain imaging GWAS data was entirely from the UKBB, we prioritized the IGAP dataset to avoid false positive results (type I error) due to sample overlap in TSMR analyses [38]. HD is an autosomal dominant disorder due to an expanded CAG trinucleotides repeat in the *HTT* gene; the data used in our MR analysis is the largest GWAS to date for the onset time of HD. All the GWASs summary data included in our study were derived from European-descent individuals to minimize the bias due to population stratification. The detailed information on GWASs for the seven major neurodegenerative diseases is provided in Supplementary Table 2.

### The estimated standardized effect size of SNPs

We standardized effect size (*β*) and standard error (*se*) in each of the seven GWAS summary statistics in order to compare effect size across studies. The estimated standardized effect size and standard error are related to minor allele frequency (MAF), sample size, original effect size, and standard error, which can be calculated by the following formula [39].

$$\beta =\frac{z}{\sqrt{2f\left(1-f)(n+{z}^{2}\right)}}$$

$${se}=\frac{1}{\sqrt{2f\left(1-f)(n+{z}^{2}\right)}}$$

where *z* can be calculated as *β/se* from the original summary data, *n* is the total sample size, and *f* is MAF.

### Bidirectional TSMR analyses

Bidirectional TSMR analyses were conducted to investigate the causal links between the 341 structural and diffusion IDPs and the seven major neurodegenerative diseases. Forward MR analyses were performed with IDPs as exposures and neurodegenerative diseases as outcomes. Conversely, reverse MR analyses were performed with neurodegenerative diseases as exposures and IDPs as outcomes.

### Selection of genetic IVs

In the forward MR analyses, SNPs strongly associated with brain IDPs (*P* < 5×10^-8^) were selected as candidate IVs. In the reverse MR analyses, SNPs strongly associated with neurodegenerative diseases (*P* < 5×10^-8^) were selected as candidate IVs. To ensure the independence of each SNP, the clumping procedure was performed in the *TwoSampleMR* R package [40], r^2^ < 0.001 and a window of 10,000 kb were used as parameters for clumping, and the 1000 Genomes European data was used as the reference panel. Palindromic SNPs with MAFs close to 0.5 were removed to avoid potential strand-flipping issues. The harmonization procedure was conducted to ensure SNPs in the exposure and outcome datasets came from the same directional DNA strand. Afterwards, SNPs associated with confounders (*P* < 5×10^-8^) were also removed from the following analyses. A number of studies support that common demographic characteristics and lifestyle risk factors have impacts on brain structures and neurodegenerative diseases. Therefore, in this study, common demographic characteristics such as socioeconomic status [41, 42] and education [43, 44], common lifestyle risk factors such as drinking [45, 46] and smoking behavior [47, 48] were included as potential confounders. PhenoScanner GWAS database V2 [49, 50] (http://www.phenoscanner.medschl.cam.ac.uk/) and NHGRI-EBI GWAS catalog [51] (https://www.ebi.ac.uk/gwas/home) were used to search SNPs associated with confounders. Finally, SNPs associated with outcomes (each neurodegenerative disease in the forward MR analyses; each IDP in the reverse MR analyses) were removed at a significant level of *P* < 5 × 10^-8^. The remaining SNPs were used for the following MR analyses.

*F* statistic was calculated to measure the strength of IVs, which is related to *R*^*2*^ (variance of exposure explained by IVs), *n* (sample size of exposure GWAS), and *k* (the number of IVs). *R*^*2*^ statistic is related to effect size (*β*) and standard error (*se*) of the exposure and *n* (sample size of exposure GWAS). *R*^*2*^ and *F* statistics can be calculated by the following formula [52],

$${R}^{2}=\frac{{\beta }^{2}}{{\beta }^{2}+{{se}}^{2}\times n}$$

$$F=\frac{{R}^{2}\times \left(n-k-1\right)}{\left(1-{R}^{2}\right)\times k}$$

The *R*^*2*^ of IVs is the sum of *R*^*2*^ of each IV. The SNPs with *F* statistic ≥10 indicated a relatively low risk of weak instrument bias and were kept for further MR analyses.

### MR analysis approaches

The inverse-variance weighted (IVW) regression [53] with multiplicative random effects was employed as the main MR analysis method, as it is the most efficient approach and accounts for heterogeneity in the variant-specific causal estimates [54]. Since the IVW method may yield a biased estimate when any of the IVs have a horizontal pleiotropy effect, we also performed the MR-Egger, weighted median (WM), maximum likelihood (ML), and MR-robust adjusted profile scores (RAPS) methods to assess the robustness of our results. The MR-Egger method can provide a consistent estimate of the causal effect under a weaker assumption even though all IVs have pleiotropy effects [55]. The WM approach can give a robust estimate of the causal effect even when up to 50% of IVs are invalid [56]. The ML method can provide an estimate with a relatively lower standard error [57]. The MR-RAPS method is robust to both systematic and idiosyncratic pleiotropy and can make a reliable causal inference with many weak IVs [58]. When only a single IV is available, the Wald ratio method [59] is used to perform causal inference. The MR-RAPS method was implemented in the “*mr.raps*” R package [58]; all other MR methods were implemented in the “*TwoSampleMR*” R package [40]. Bonferroni correction was applied to control for multiple testing. The effective number of independent tests was calculated using matrix spectral decomposition (matSpD) approach [60, 61]. Pearson correlation matrix was computed and inputted to the *matSpD.R* script (https://drive.google.com/open?id=1-r-HWsKOD8NfbOG4C4SFIwjj8yYze2Zu). The effective number of independent variables is 135.48 for the 341 IDPs. After considered the kinds of disease and forward and reverse MR analyses, the final Bonferroni correction level was set to *P* < 2.64 × 10^−5^ (0.05/135.48/7/2). Meanwhile, *P* < 1 × 10^−4^ was set as nominal significance.

### Sensitivity analysis

The significant results detected by the IVW estimate were further verified by a set of sensitivity analyses. First, Cochran’s Q test [62] was used to measure the heterogeneity between variant-specific causal estimates due to pleiotropy or other causes. Second, MR Pleiotropy RESidual Sum and Outlier (MR-PRESSO) approach [63] was employed to evaluate the horizontal pleiotropy. Third, MR-Egger intercept test [64] was performed to detect the potential directional pleiotropy. Fourth, leave-one-out sensitivity test (i.e., drop one IV from the analysis and re-estimate the causal effect) were conducted to assess if the MR estimate is driven by a single SNP. The Cochran’s Q test, MR-Egger intercept test, and leave-one-out analyses were performed using the “*TwoSampleMR*” R package [40] and the MR-PRESSO approach was performed using the “*MR-PRESSO*” R package [63]. In addition, we included common diseases such as obesity [65, 66] and hypertension [67, 68] as confounding factors and reperformed the MR analyses, and the results are presented in Supplementary Tables 13, 14. We also performed MR analyses using the IVs excluding SNPs within human major histocompatibility complex (MHC) since genetic variants in MHC regions are known to be associated with numerous complex human diseases [69]. These results are presented in Supplementary Tables 15, 16. What’s more, we used a stricter variants selection threshold (*P* < 5 × 10^−9^) and re-executed the MR analysis for sensitivity analysis (Supplementary Tables 17, 18). We also relaxed the threshold (*P* < 5 × 10^-6^ and *F* > 10) when the IVs were fewer than 4 and re-performed the MR analyses as part of sensitivity analysis (Supplementary Tables 19, 20).

### Power calculation

An online public tool (https://sb452.shinyapps.io/power/) [70] provided by Burgess was applied to estimate statistical power for all the significant MR analysis results. Briefly, for the continuous outcome, the parameters required to calculate power are the GWAS sample size of the outcome, explained variance of IVs on the exposure, and causal effect of MR analysis (change in standard deviation [SD] in outcome per SD change in exposure); for the binary outcome, the parameters required to calculate power are the GWAS sample size of the outcome, explained variance of IVs on the exposure, case-control ratio of each neurodegenerative disease, and causal effect of MR analysis (odds ratio [OR] per SD change in exposure).

## Results

### Genetic IVs selection and instrument strength

A total of 74 SNPs in forward MR and 4 SNPs in reverse MR analyses associated with confounders were removed (Supplementary Tables 3 and 4). The *F* statistics of all IVs were ≥ 10, which suggests a low risk of weak instrument bias [52]. SNPs kept for the final bidirectional TSMR analyses are presented in Supplementary Tables 5, 6.

### Causal effects of IDPs on neurodegenerative diseases

**Fig. 2:**
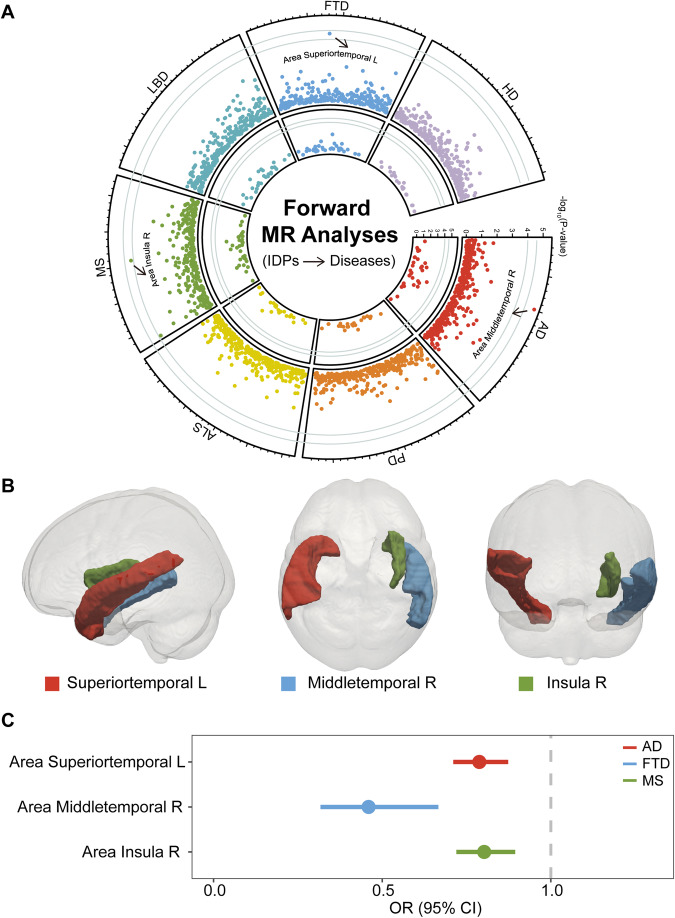
The results of forward MR analyses (brain IDPs as exposures and neurodegenerative diseases as outcomes). **A** Circos plot shows all the results. The x-axis shows each IDP and the y-axis shows -log_10_(*P*) values. The outer track represents the results in the IVW method and inner track represents the results in the Wald ratio method. The outer and inner gray lines represent *P* = 2.64×10^-5^ and *P* = 1.00×10^-4^, respectively. **B** Pattern diagram shows the brain locations of the significant and nominal results of the causal effects of brain IDPs on AD, FTD, and MS. **C** Forest plot shows significant and nominally significant results of the causal effects of brain IDPs on AD, FTD, and MS in the IVW method. The x-axis shows OR (odds ratio) and 95% CI (confidence interval). Please refer to Fig. 1 legends for all the abbreviations.

The putative causal relationships between three structural IDPs and three neurodegenerative diseases (AD, FTD, and MS) were identified in the forward MR analyses. As shown in Fig. 2 and Supplementary Table 7, the IVW estimates suggested that genetically predicted SA of the left superior temporal gyrus was significantly negatively associated with AD risk [OR = 0.79; 95% confidence interval (CI), 0.71–0.87; *P* = 5.99 × 10^−6^]. The IVW estimates also showed that genetically predicted SA of the right middle temporal gyrus was nominally negatively associated with FTD risk (OR = 0.46; 95% CI, 0.32–0.67; *P* = 4.17 × 10^−5^) and genetically predicted SA of the right insula was nominally negatively associated with MS risk (OR = 0.80; 95% CI, 0.72–0.89; *P* = 6.85 × 10^−5^).

### Causal effects of neurodegenerative diseases on IDPs

**Fig. 3:**
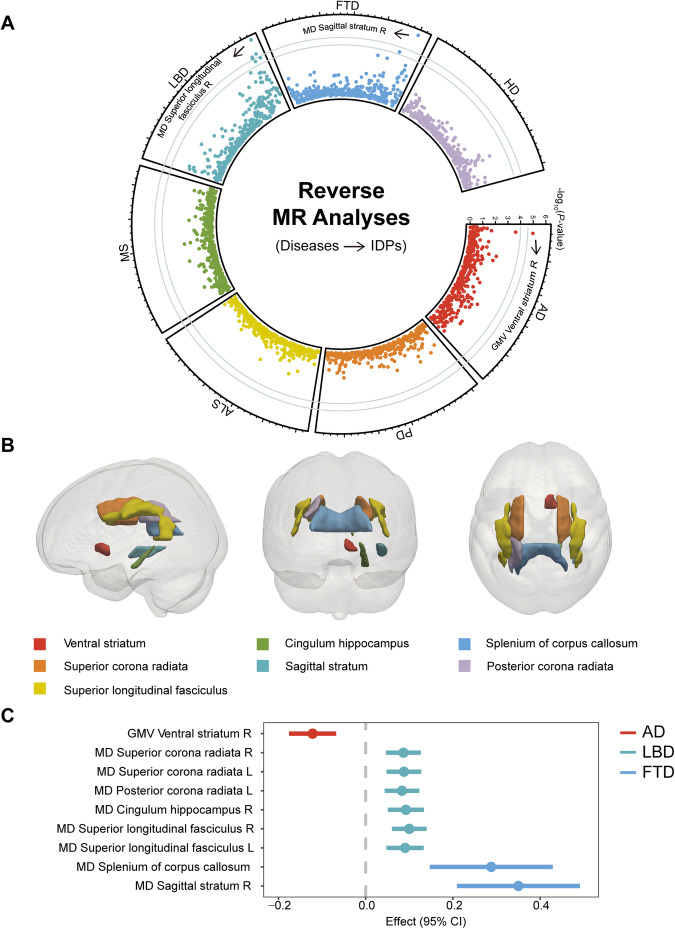
The results of reverse MR analyses (neurodegenerative diseases as exposures and brain IDPs as outcomes). **A** Circos plot shows all the results. The x-axis shows each IDP and the y-axis shows -log_10_(*P*) values. The results of FTD are present in the Wald ratio method and the results of other diseases are present in the IVW method. The outer and inner gray lines represent *P* = 2.64 × 10^−5^ and *P* = 1.00 × 10^−4^, respectively. **B** Pattern diagram shows the brain locations of the significant and nominal results of the causal effects of brain IDPs on AD, LBD, and FTD. **C** Forest plot shows significant and nominally significant results of the causal effects of AD, LBD, and FTD on brain IDPs in the IVW or Wald ratio method. The x-axis shows causal effects and 95% CI. Please refer to Fig. 1 legends for all the abbreviations.

The putative causal effects of AD, LBD, and FTD on nine structural and diffusion IDPs were found in reverse MR analyses. The IVW estimates suggested that genetically predicted AD was significantly correlated with the decreased GMV in the right ventral striatum (*β* = −0.12; 95% CI, −0.18 to −0.07; *P* = 1.03 × 10^−5^). Moreover, the IVW estimates demonstrated that genetically predicted LBD was associated with increased MD in diverse projection and association fibres, which reflected microstructural damage of white matter connections (Fig. 3). Specifically, the MD in right superior longitudinal fasciculus (SLF, *β* = 0.10; 95% CI, 0.06 to 0.14; *P* = 8.80 × 10^−7^), right cingulum in the hippocampus (CgH, *β* = 0.09; 95% CI, 0.02 to 0.05; *P* = 1.33 × 10^−5^), left superior corona radiata (SCR, *β* = 0.09; 95% CI, 0.05 to 0.13; *P* = 1.64 × 10^−5^) and right SCR (*β* = 0.09; 95% CI, 0.05 to 0.13; *P* = 2.09 × 10^−5^) were significantly increased by LBD. In addition, genetically predicted FTD was found to be significantly associated with increased MD in the right sagittal stratum (SS, *β* = 0.35; 95% CI, 0.21 to 0.49; *P* = 1.13 × 10^−6^) in Wald ratio method (only a single IV was available). Genetically predicted LBD was nominally associated with increased MD in left SLF (*β* = 0.09; 95% CI, 0.05 to 0.13; *P* = 3.52 × 10^−5^) and left posterior corona radiata (PCR, *β* = 0.08; 95% CI, 0.04 to 0.12; *P* = 4.36 × 10^−5^) and FTD was nominally associated with increased MD in splenium of corpus callosum (SCC, *β* = 0.29; 95% CI, 0.15–0.43; *P* = 6.32 × 10^−5^). The detailed information is shown in Fig. 3 and Supplementary Table 8.

### Sensitivity analyses

The effect directions of the estimates using the MR-Egger, WM, ML, and MR-RAPS methods were all in line with those using the IVW method. However, only a few results supported the IVW method remained significant in four supplementary MR methods. Specifically, the numbers of associations using ML, MR-RAPS, WM, and MR-Egger methods were 8, 8, 6, and 0, respectively, by using the same threshold (*P* < 1 × 10^−4^, 10 associations in the IVW methods). The inconsistency could potentially be attributed to the relatively lower statistical power of these four supplementary MR methods compared with that of the IVW method [54]. The detailed results are shown in Supplementary Tables 7-8.

Cochran’s Q test indicated that there was no significant heterogeneity between IVs (*P* > 0.05). The MR-PRESSO and MR-Egger intercept tests indicated no significant horizontal pleiotropy or outliers. Leave-one-out analyses suggested that the results of MR estimates were not driven by a single SNP except the causal effect of LBD on several DTI metrics. The detailed results of sensitivity analyses are shown in Supplementary Tables 9 and 10. The power of significant results in our MR analyses ranged from 46.6% to 99.8%. Similar results of forward and reverse MR analyses were found before and after removing confounders in the IVW method (Supplementary Tables 11-12).

## Discussion

In our study, we conducted bidirectional TSMR analyses to investigate the causal relationships between 341 widely used structural and diffusion IDPs and seven major neurodegenerative diseases. We identified the putative causal effects of three IDPs on AD, FTD, and MS, and the putative causal effects of AD, FTD, and LBD on nine IDPs.

AD is characterized by progressive memory loss and cognitive impairment. In the forward MR analyses, a genetically predicted small area of the left superior temporal gyrus was correlated with the increased risk of AD. The human superior temporal gyrus is critical for speech comprehension [71], while the speech comprehension ability is damaged in AD patients. Significant atrophy of the bilateral superior temporal gyrus in AD patients has been reported in a recent case-control survey [72]. Our result expands the findings of the observational study and suggests that atrophy of the superior temporal gyrus is a potential causal factor of AD. The reverse MR analyses revealed a statistical causal effect of genetically predicted AD on the reduction of GMV in the right ventral striatum. The ventral striatum is a prominent brain structure regulating reward-oriented behaviour [73]. Previous studies have shown that neuropsychiatric symptoms including reduction of reward-oriented behaviour are common in AD patients [74]. Meanwhile, atrophy of the ventral striatum in AD has been reported in an observational neuroimaging study [75]. Our result may contribute to understanding the neural mechanisms of neuropsychiatric symptoms in patients with AD. Our results were not completely consistent with previous MR studies. For example, causal relationships between brain IDPs and AD reported by Song et al.’s [25] and Wu et al.’s [26] were not observed in our study, and we reported two new statistical causal associations between structural IDPs and AD. Several reasons might result in this inconsistency. First, the GWAS summary datasets of brain IDPs and AD selected by our study and previous studies are somewhat different. For instance, compared to Song et al.’s [25] and Wu et al.’s [26], our study selected the GWAS summary statistics of AD that did not include the AD-by-proxy from the UKBB. While, all GWAS summary datasets of AD and IDPs used by Song et al.’s [25] and Wu et al.’s [26] studies comprised the AD-by-proxy from the UKBB, which led to increased heterogeneity in data and sample overlap between the exposure and the outcome and thus introduced bias in MR analyses. Second, we selected the latest release of the UKBB imaging study with the most comprehensive brain IDPs, the number of the brain IDPs chosen in our study is much larger than in previous studies. Third, due to the numbers of IDPs and neurodegenerative diseases selected in our study and previous studies being different, the *P* value thresholds for statistical significance were different. Fourth, we removed SNPs associated with several confounders having impacts on brain structure and neurodegenerative diseases, which might influence the results of MR analyses.

FTD is a heterogeneous neurodegenerative disorder comprising three clinical variants: behavioural-variant FTD (bvFTD), non-fluent variant primary progressive aphasia (nvPPA), and semantic-variant primary progressive aphasia (svPPA) [76]. In the forward MR analyses, genetically predicted small SA of the right middle temporal gyrus was correlated with the increased risk of FTD. Atrophy of the frontal, temporal, and insular lobes is common in FTD, and the pattern of atrophy is usually asymmetric [14]. Our results extended these findings by identifying the potential causal relationship between the atrophy of the right temporal lobe and FTD. In the reverse MR analyses, genetically predicted FTD was found to be associated with increased MD in right SS and SCC. The SCC is the most posterior portion of the corpus callosum containing numerous crossing fibres that mainly connect bilateral temporal, posterior parietal, and occipital cortices. The SCC is mainly associated with visuospatial information transfer, language, reading, and calculation scores [77]. Numerous studies have shown the damage of SCC in FTD patients [78, 79]. The SS is a large, sheet-like, sagittal structure within the deep white matter of the occipital, temporal, and parietal lobes. Fibres running in the SS are still unclear; the optic radiation and inferior longitudinal fasciculus may be the main components of SS [80]. Although the alteration of SS in FTD patients has not been reported, observational studies have demonstrated the damage of inferior longitudinal fasciculus in FTD patients [81]. Our findings yield insights into the neural mechanisms of FTD and functional impairments in FTD patients.

MS is a central nervous system demyelinating and neurodegenerative disease with varying presentation, disease courses, and prognosis. In the forward MR analyses, we found a suggestively statistically negative causal effect of the genetically predicted SA of the right insula on MS risk. The insula is implicated in affection as well as a diverse range of cognitive functions [82]. Previous observational studies have revealed that the atrophy of insula is associated with cognitive impairment and fatigue in MS patients [83]. Our findings may contribute to the understanding of the causal association between insula atrophy and MS and help to develop novel biomarkers of MS.

LBD is characterised by fluctuating cognitive decline, visual hallucinations, parkinsonism, and sleep disturbance [84]. In the reverse MR analyses, genetically predicted LBD was found to be associated with microstructural damage of SLF, SCR, PCR, and CgH. The corona radiata includes the thalamic radiations (thalamocortical, corticothalamic fibres) and parts of the long corticofugal pathways, such as the corticospinal, corticopontine, and corticobulbar tracts. An observational MRI study revealed that white matter tracts connecting the brain cortex and thalamus were damaged in LBD patients with hallucinations compared to patients without hallucinations [85]. The SLF tract is an association fibre located at the dorsolateral regions of the corona radiata and contains connections between the ipsilateral frontal, parietal, occipital, and temporal lobes. The cingulum carries information from the cingulate gyrus to the hippocampus, and the CgH is the hippocampal formation part of the cingulum. Damage in SLF [86] and cingulum [87] in LBD patients has also been reported by observational studies. Our results were in part consistent with previous findings and could deepen the understanding of the microstructural damage of white matter tracts in LBD progression.

In this study, we investigated the causal relationships between brain IDPs and seven major neurodegenerative diseases using the TSMR approach. Apart from AD and ALS, there have been no investigations exploring the causal association between brain IDPs and the remaining five neurodegenerative diseases (PD, MS, LBD, FTD, and HD). In the forward MR analyses, we reported putative causal associations between two IDPs and FTD and MS. In the reverse MR analyses, we reported putative causal associations between LBD and FTD and eight IDPs. All of the above were reported here for the first time. In this study, we reported two novel putative causal associations between brain IDPs and AD. Although there were four studies exploring the causal relationship between brain IDPs and AD [25–28], two of which reported suggestive causal associations, our findings still remain meaningful. Our results are more reliable since we utilized more homogeneous AD GWAS data [20] and a large-sample GWAS data of comprehensive brain IDPs [24]. Recently, Wang and colleagues [88] investigated the causal relationship between brain IDPs and ALS, revealing that a genetically determined high orientation dispersion index in the right cerebral peduncle was associated with an increased risk of ALS. However, this study utilized a relatively small ALS GWAS dataset (20,806 cases/59,804 controls) and employed overly lenient clumping methods (r^2^ < 0.1 within a window of 500 kb) for selecting independent SNPs for the exposure, which may lead to a higher probability of type 1 error. In contrast, we utilized a larger ALS GWAS dataset (27,205 cases/110,881 controls) and implemented a more stringent IVs selection procedure (r^2^ < 0.001 within a window of 10,000 kb), yet failed to replicate the findings of Wang et al. [88] (OR = 1.06, 95% CI = 0.96–1.16, *P* = 0.24). Therefore, the result between the brain IDP and ALS reported by Wang et al. [88] should be interpreted with caution.

There are several limitations should be addressed in the current study. First, in this TSMR investigation, we attempted to make causal inferences between structural and diffusion IDPs and major neurodegenerative diseases by using SNPs associated with the exposure as IVs. However, although we have conducted a series of rigorous genetic IVs selection steps, we can never eliminate intrinsic uncertainty in the instrumental variable assumptions, which means that there remains somewhat uncertainty in the putative causal conclusion. For instance, although we have removed SNPs associated with certain confounders in the genetic IVs selection, we cannot take all potential confounders in consideration. Second, MR is only a statistical method that uses genetic associations with both the putative causal factor (i.e., exposure) and the outcome to infer possible causality. The MR findings reflect differences across the life course in a risk factor, not the effects at a specific time after an intervention is implemented. It should therefore be treated with caution when applied to clinical intervention. Third, because the participants of the datasets included here are all of European descent, the statistical causal associations cannot be generalized to other ethnicities and races. Fourth, due to the lack of detailed information of each individual, we are not able to estimate how well the age distributions of disease GWAS match the IDP GWAS. Finally, The UKBB study is well known for its “healthy volunteer” selection bias [89] and may not be representative of the general population.

In conclusion, we identified statistical causal associations between several structural and diffusion IDPs and neurodegenerative diseases using bidirectional TSMR analyses, which might contribute to clarifying the etiology of these diseases and the influences of these diseases on brain structure. In addition, our findings might also promote the early detection and prevention of neurodegenerative diseases at the neuroimaging level. The three structural IDPs identified in the forward MR analyses could be potential biomarkers for detecting high-risk individuals of AD, FTD, and MS. The structural and diffusion IDPs identified in the reverse MR analyses could be potential biomarkers for explaining functional impairments and potential intervention targets of AD, FTD, and LBD patients.

## Data and code availability

GWAS summary statistics of IDPs and seven major neurodegenerative diseases were publicly available [20–22, 24, 30–33]. All the detailed data and R language codes used for conducting the analysis in our study are available at https://github.com/ziruiwang313/IDP_NDD_MR.git.

### Supplementary information

Supplementary Materials

Supplementary Table S1

Supplementary Table S2

Supplementary Table S3

Supplementary Table S4

Supplementary Table S5

Supplementary Table S6

Supplementary Table S7

Supplementary Table S8

Supplementary Table S9

Supplementary Table S10

Supplementary Table S11

Supplementary Table S12

Supplementary Table S13

Supplementary Table S14

Supplementary Table S15

Supplementary Table S16

Supplementary Table S17

Supplementary Table S18

Supplementary Table S19

Supplementary Table S20

Supplementary Figure 1

Supplementary Figure 2

Supplementary Figure 3

Supplementary Figure 4
