# Supplementary material for: Bidirectional two-sample Mendelian randomization analyses support causal relationships between structural and diffusion imaging-derived phenotypes and the risk of major neurodegenerative diseases
Source: Transl Psychiatry. 2024 May 28;14:215. doi: 10.1038/s41398-024-02939-3 (PMC11133432; doi:10.1038/s41398-024-02939-3)
Supplement: Supplementary file 1 — Supplementary Materials [file 41398_2024_2939_MOESM1_ESM.docx]

**Supplementary Materials**

**UK Biobank (UKBB) resource**

UKBB is a prospective study that aims to assess the relevance of a wide range of exposures to a wide range of health-related outcomes[1]. Over 500,000 participants aged 40-69 years have been recruited in 2006-2010 from all around the UK. Extensive genetic and phenotypic information about participants, including data from questionnaires, physical measures, sample assays, accelerometry, multimodal imaging, and genome-wide genotyping were collected, and a diverse set of health-related outcomes (including cancer, heart disease, stroke, diabetes, dementia, etc.) are followed up.

Although brain imaging is nowadays predominantly used in the diagnosis and monitoring of disease progression, it has been found to have huge potential for early disease prediction. The objective of the UKBB imaging study was to create an extensive brain magnetic resonance imaging (MRI) dataset encompassing 100,000 healthy participants, thereby helping the discovery of novel imaging biomarkers predicting the initiation and progression of diverse diseases[2]. The MRI data modalities of UKBB comprise structural, diffusion and functional MRI, which can reflect the anatomical and neuropathological structure, local tissue microstructure, and brain activity, respectively. All brain MRI images were acquired using a Siemens Skyra 3 T scanner with a standard 32-channel radiofrequency receive head coil. T1 structural images were obtained using a 3D magnetization-prepared rapid gradient-echo (MPRAGE) sequence with the following parameters: resolution = 1 × 1 × 1 mm, field-of-view = 208 × 256 × 256 matrix, duration = 5 minutes. The diffusion-weighted images were obtained with the following parameters: resolution = 2 × 2 × 2 mm, field-of-view = 104 × 104 × 72 matrix, duration = 7 minutes, two shells of b = 1000 and 2000 s/mm^2^ with 50 direction per shell, fat saturation. The details of the scanning parameters of all UKBB MRI modalities can be found at: biobank.ctsu.ox.ac.uk/crystal/docs/brain_mri.pdf. The raw MRI data were processed automatically to produce both processed images as well as thousands of image-derived phenotypes (IDPs)[3].

For the T1-derived structural IDPs, we selected the most commonly used metrics: gray matter volume (GMV, unit: mm^3^), surface area (SA, unit: mm^2^), and cortical thickness (CT, unit: mm), which can measure the volumes and morphology of brain tissues and structures from different dimensions. Global or local GMV loss is a sensitive biomarker of neurodegenerative diseases[4,5]. Alterations of brain surface area and cortical thickness have also been found to be associated with function impairment in neurodegenerative diseases[6].

Diffusion MRI measures the random movement of water molecules (water diffusion) within their microscopic tissue environment. For the diffusion IDPs, we selected the most commonly used measures: fractional anisotropy (FA) and mean diffusivity (MD, unit: mm^2^/s). FA measures the degree of diffusion directionality with values ranging from 0 (isotropic diffusion) to 1 (totally anisotropic diffusion). FA is associated with the micro-structure of the brain, such as axonal diameter, axonal density, and degree of myelination[7]. MD measures the average magnitude of water diffusion within brain tissue and is a rather non-specific metric. Decreased FA and increased MD values may reflect the deviations in the integrity of white matter tracts. Alterations of FA and MD in diverse brain regions in neurodegenerative diseases have been identified by a number of observational studies[4,8].

**Supplementary Table S21.** STROBE-MR checklist of recommended items to address in reports of Mendelian randomization studies.

| **Item No.** | **Section** | **Checklist item** | **Page No.** |
| --- | --- | --- | --- |
| 1 | TITLE and ABSTRACT | Indicate Mendelian randomization (MR) as the study’s design in the title and/or the abstract if that is a main purpose of the study | 1;3 |
|  | INTRODUCTION |  |  |
| 2 | Background | Explain the scientific background and rationale for the reported study. What is the exposure? Is a potential causal relationship between exposure and outcome plausible? Justify why MR is a helpful method to address the study question | 4-6 |
| 3 | Objectives | State specific objectives clearly, including pre-specified causal hypotheses (if any). State that MR is a method that, under specific assumptions, intends to estimate causal effects | 7 |
|  | METHODS |  |  |
| 4 | Study design and data sources | Present key elements of the study design early in the article. Consider including a table listing sources of data for all phases of the study. For each data source contributing to the analysis, describe the following: |  |
|  | a) | Setting: Describe the study design and the underlying population, if possible. Describe the setting, locations, and relevant dates, including periods of recruitment, exposure, follow-up, and data collection, when available. | 8-9 |
|  | b) | Participants: Give the eligibility criteria, and the sources and methods of selection of participants. Report the sample size, and whether any power or sample size calculations were carried out prior to the main analysis | 8-9;14 |
|  | c) | Describe measurement, quality control and selection of genetic variants | 10-11 |
|  | d) | For each exposure, outcome, and other relevant variables, describe methods of assessment and diagnostic criteria for diseases | 8-9 |
|  | e) | Provide details of ethics committee approval and participant informed consent, if relevant | 14 |
| 5 | Assumptions | Explicitly state the three core IV assumptions for the main analysis (relevance, independence and exclusion restriction) as well assumptions for any additional or sensitivity analysis | 13 |
| 6 | Statistical methods: main analysis | Describe statistical methods and statistics used |  |
|  | a) | Describe how quantitative variables were handled in the analyses (i.e., scale, units, model) | 10 |
|  | b) | Describe how genetic variants were handled in the analyses and, if applicable, how their weights were selected | 10-11 |
|  | c) | Describe the MR estimator (e.g. two-stage least squares, Wald ratio) and related statistics. Detail the included covariates and, in case of two-sample MR, whether the same covariate set was used for adjustment in the two samples | 12-13 |
|  | d) | Explain how missing data were addressed | 12-13 |
|  | e) | If applicable, indicate how multiple testing was addressed | 12 |
| 7 | Assessment of assumptions | Describe any methods or prior knowledge used to assess the assumptions or justify their validity | 4-6 |
| 8 | Sensitivity analyses and additional analyses | Describe any sensitivity analyses or additional analyses performed (e.g. comparison of effect estimates from different approaches, independent replication, bias analytic techniques, validation of instruments, simulations) | 13 |
| 9 | Software and pre-registration |  |  |
|  | a) | Name statistical software and package(s), including version and settings used | 12 |
|  | b) | State whether the study protocol and details were pre-registered (as well as when and where) | NA |
|  | RESULTS |  |  |
| 10 | Descriptive data |  |  |
|  | a) | Report the numbers of individuals at each stage of included studies and reasons for exclusion. Consider use of a flow diagram | 15 |
|  | b) | Report summary statistics for phenotypic exposure(s), outcome(s), and other relevant variables (e.g. means, SDs, proportions) | 15-16 |
|  | c) | If the data sources include meta-analyses of previous studies, provide the assessments of heterogeneity across these studies | NA |
|  | d) | For two-sample MR:  i. Provide justification of the similarity of the genetic variant-exposure associations between the exposure and outcome samples  ii. Provide information on the number of individuals who overlap between the exposure and outcome studies | Supplementary materials |
| 11 | Main results |  |  |
|  | a) | Report the associations between genetic variant and exposure, and between genetic variant and outcome, preferably on an interpretable scale | 15-16 |
|  | b) | Report MR estimates of the relationship between exposure and outcome, and the measures of uncertainty from the MR analysis, on an interpretable scale, such as odds ratio or relative risk per SD difference | 15-16 |
|  | c) | If relevant, consider translating estimates of relative risk into absolute risk for a meaningful time period | 15-16 |
|  | d) | Consider plots to visualize results (e.g. forest plot, scatterplot of associations between genetic variants and outcome versus between genetic variants and exposure) | Supplementary materials |
| 12 | Assessment of assumptions |  |  |
|  | a) | Report the assessment of the validity of the assumptions | 15-16 |
|  | b) | Report any additional statistics (e.g., assessments of heterogeneity across genetic variants, such as I2, Q statistic or E-value) | 15-16 |
| 13 | Sensitivity analyses and additional analyses |  |  |
|  | a) | Report any sensitivity analyses to assess the robustness of the main results to violations of the assumptions | 16-17 |
|  | b) | Report results from other sensitivity analyses or additional analyses | 16-17 |
|  | c) | Report any assessment of direction of causal relationship (e.g., bidirectional MR) | 15-16 |
|  | d) | When relevant, report and compare with estimates from non-MR analyses | NA |
|  | e) | Consider additional plots to visualize results (e.g., leave-one-out analyses) | 17 |
|  | DISCUSSION |  |  |
| 14 | Key results | Summarize key results with reference to study objectives | 17 |
| 15 | Limitations | Discuss limitations of the study, taking into account the validity of the IV assumptions, other sources of potential bias, and imprecision. Discuss both direction and magnitude of any potential bias and any efforts to address them | 21-22 |
| 16 | Interpretation |  |  |
|  | a) | Meaning: Give a cautious overall interpretation of results in the context of their limitations and in comparison with other studies | 17-20 |
|  | b) | Mechanism: Discuss underlying biological mechanisms that could drive a potential causal relationship between the investigated exposure and the outcome, and whether the gene-environment equivalence assumption is reasonable. Use causal language carefully, clarifying that IV estimates may provide causal effects only under certain assumptions | 17-20 |
|  | c) | Clinical relevance: Discuss whether the results have clinical or public policy relevance, and to what extent they inform effect sizes of possible interventions | 17-20 |
| 17 | Generalizability | Discuss the generalizability of the study results (a) to other populations, (b) across other exposure periods/timings, and (c) across other levels of exposure | 17-20 |
|  | OTHER INFORMATION |  |  |
| 18 | Funding | Describe sources of funding and the role of funders in the present study and, if applicable, sources of funding for the databases and original study or studies on which the present study is based | 23 |
| 19 | Data and data sharing | Provide the data used to perform all analyses or report where and how the data can be accessed and reference these sources in the article. Provide the statistical code needed to reproduce the results in the article, or report whether the code is publicly accessible and if so, where | 14 |
| 20 | Conflicts of Interest | All authors should declare all potential conflicts of interest | 23 |

**Supplementary Figure legends**

**Supplementary Figure 1 The scatter plots of significant** **and nominal results of bidirectional MR analyses.** **(A)** SA of the left superior temporal gyrus on AD. **(B)** SA of the right middle temporal gyrus on FTD. **(C)** SA of the right insula on MS. **(D)** AD on GMV of the right ventral striatum. **(E)** LBD on MD of the right superior corona radiata. **(F)** LBD on MD of the left superior corona radiata. **(G)** LBD on MD of the left posterior corona radiata. **(H)** LBD on MD of the right cingulum hippocampus. **(I)** LBD on MD of the right superior longitudinal fasciculus. **(J)** LBD on MD of the left superior longitudinal fasciculus. Abbreviation: AD, Alzheimer’s disease; FTD, frontotemporal dementia; GMV, gray matter volume; LBD, Lewy body dementia; MD, mean diffusivity; MS, multiple sclerosis; SA, surface area. Please refer to Figure S1 legends for the all the abbreviations.

**Supplementary Figure 2 The leave-one-out plots of significant** **and nominal results of bidirectional MR analyses.** **(A)** SA of the left superior temporal gyrus on AD. **(B)** SA of the right middle temporal gyrus on FTD. **(C)** AD on GMV of the right ventral striatum. **(D)** LBD on MD of the right superior corona radiata. **(E)** LBD on MD of the left superior corona radiata. **(F)** LBD on MD of the left posterior corona radiata. **(G)** LBD on MD of the right cingulum hippocampus. **(H)** LBD on MD of the right superior longitudinal fasciculus. **(I)** LBD on MD of the left superior longitudinal fasciculus. Please refer to Figure S1 legends for the all the abbreviations.

**Supplementary Figure 3 The forest plots of significant** **and nominal results of bidirectional MR analyses.** **(A)** SA of the left superior temporal gyrus on AD. **(B)** SA of the right middle temporal gyrus on FTD. **(C)** SA of the right insula on MS. **(D)** AD on GMV of the right ventral striatum. **(E)** LBD on MD of the right superior corona radiata. **(F)** LBD on MD of the left superior corona radiata. **(G)** LBD on MD of the left posterior corona radiata. **(H)** LBD on MD of the right cingulum hippocampus. **(I)** LBD on MD of the right superior longitudinal fasciculus. **(J)** LBD on MD of the left superior longitudinal fasciculus. Please refer to Figure S1 legends for the all the abbreviations.

**Supplementary Figure 4 The funnel plots of significant** **and nominal results of bidirectional MR analyses.** **(A)** SA of the left superior temporal gyrus on AD. **(B)** SA of the right middle temporal gyrus on FTD. **(C)** SA of the right insula on MS. **(D)** AD on GMV of the right ventral striatum. **(E)** LBD on MD of the right superior corona radiata. **(F)** LBD on MD of the left superior corona radiata. **(G)** LBD on MD of the left posterior corona radiata. **(H)** LBD on MD of the right cingulum hippocampus. **(I)** LBD on MD of the right superior longitudinal fasciculus. **(J)** LBD on MD of the left superior longitudinal fasciculus. Please refer to Figure S1 legends for the all the abbreviations.

**Supplementary Table legends**

**Supplementary Table 1** Detailed information on GWAS summary-level data of brain IDPs.

**Supplementary Table 2** Detailed information on GWAS summary-level data of neurodegenerative diseases.

**Supplementary Table 3** Information of IVs associated with confounders in forward MR analyses.

**Supplementary Table 4** Information of IVs associated with confounders in reverse MR analyses.

**Supplementary Table 5** Information of IVs for all exposure-outcome pairs in forward MR analyses.

**Supplementary Table 6** Information of IVs for all exposure-outcome pairs in reverse MR analyses.

**Supplementary Table 7** Forward MR analysis results.

**Supplementary Table 8** Reverse MR analysis results.

**Supplementary Table 9** MR Sensitivity analyses for the significant results of forward MR analyses.

**Supplementary Table 10** MR Sensitivity analyses for the significant results of reverse MR analyses.

**Supplementary Table 11** Comparing the IVW results before and after confounder filtering in forward MR analyses.

**Supplementary Table 12** Comparing the IVW results before and after confounder filtering in reverse MR analyses.

**Supplementary Table 13** Forward MR analysis results after excluding IVs within MHC.

**Supplementary Table 14** Reverse MR analysis results after excluding IVs within MHC.

**Supplementary Table 15** Forward MR analysis results after excluding IVs associated with additional two confounders.

**Supplementary Table 16** Reverse MR analysis results after excluding IVs associated with additional two confounders.

**Supplementary Table 17** Forward MR analysis results using variants selection threshold at *P* < 5×10^-9^.

**Supplementary Table 18** Reverse MR analysis results variants selection threshold at *P* < 5×10^-9^.

**Supplementary Table 19** Forward MR analysis results after relaxing the variants selection at *P* < 5×10^-6^ and *F* > 10 when IVs less than 4.

**Supplementary Table 20** Reverse MR analysis results after relaxing the variants selection threshold at *P* < 5×10^-6^ and *F* > 10 when IVs less than 4.

References

1 Sudlow C, Gallacher J, Allen N, Beral V, Burton P, Danesh J, et al. UK biobank: an open access resource for identifying the causes of a wide range of complex diseases of middle and old age. PLoS Med. 2015;12(3):e1001779.

2 Miller KL, Alfaro-Almagro F, Bangerter NK, Thomas DL, Yacoub E, Xu J, et al. Multimodal population brain imaging in the UK Biobank prospective epidemiological study. Nat Neurosci. 2016;19(11):1523-36.

3 Alfaro-Almagro F, Jenkinson M, Bangerter NK, Andersson JLR, Griffanti L, Douaud G, et al. Image processing and Quality Control for the first 10,000 brain imaging datasets from UK Biobank. Neuroimage. 2018;166:400-24.

4 Chandra A, Dervenoulas G, Politis M. Magnetic resonance imaging in Alzheimer's disease and mild cognitive impairment. J Neurol. 2019;266(6):1293-302.

5 Yousaf T, Dervenoulas G, Valkimadi P-E, Politis M. Neuroimaging in Lewy body dementia. J Neurol. 2019;266(1).

6 Wei X, Wang Z, Zhang M, Li M, Chen Y-C, Lv H, et al. Brain Surface Area Alterations Correlate With Gait Impairments in Parkinson's Disease. Front Aging Neurosci. 2022;14:806026.

7 Taki Y, Thyreau B, Hashizume H, Sassa Y, Takeuchi H, Wu K, et al. Linear and curvilinear correlations of brain white matter volume, fractional anisotropy, and mean diffusivity with age using voxel-based and region-of-interest analyses in 246 healthy children. Hum Brain Mapp. 2013;34(8):1842-56.

8 Kalra S, Müller H-P, Ishaque A, Zinman L, Korngut L, Genge A, et al. A prospective harmonized multicenter DTI study of cerebral white matter degeneration in ALS. Neurology. 2020;95(8):e943-e52.
