# Supplementary figures and images for: Bidirectional two-sample Mendelian randomization analyses support causal relationships between structural and diffusion imaging-derived phenotypes and the risk of major neurodegenerative diseases
Source: Transl Psychiatry. 2024 May 28;14:215. doi: 10.1038/s41398-024-02939-3 (PMC11133432; doi:10.1038/s41398-024-02939-3)

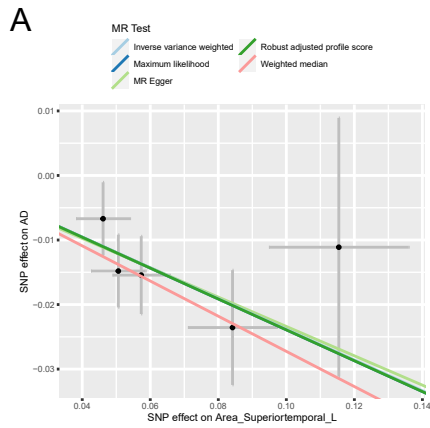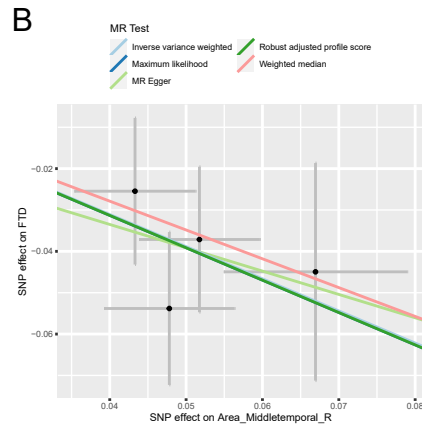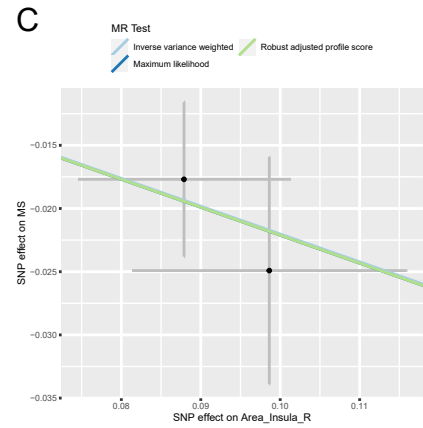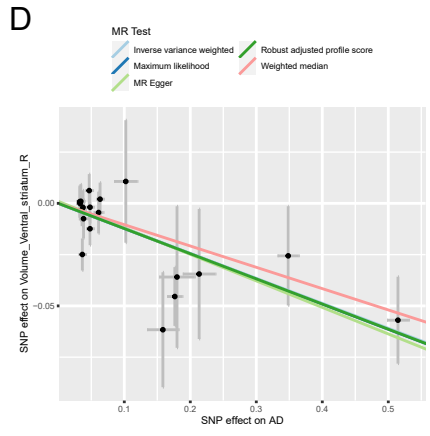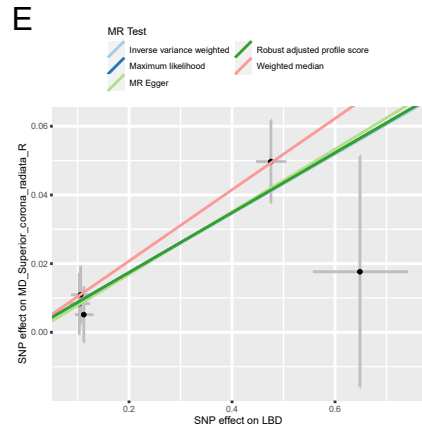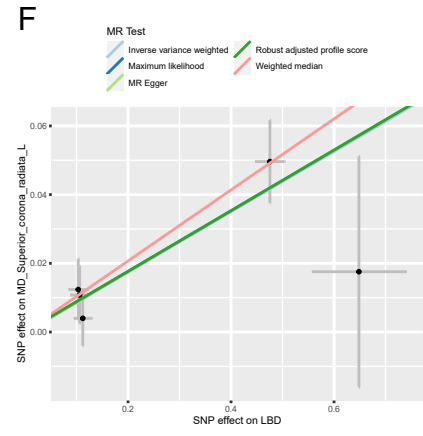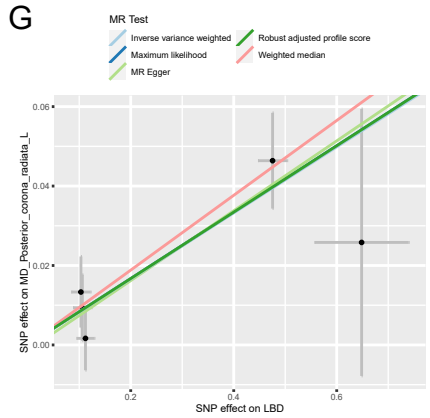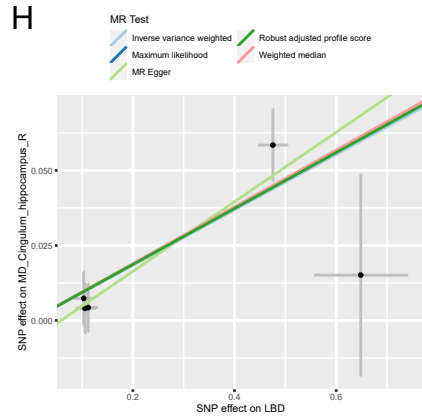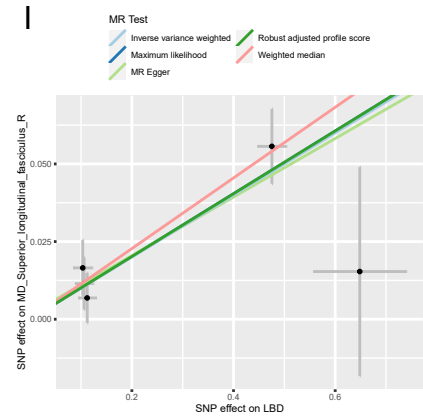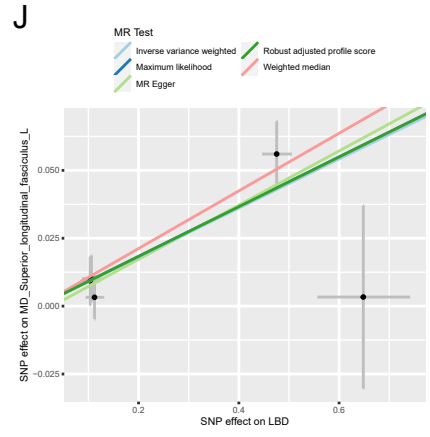

Supplement: Supplementary file 22 — Supplementary Figure 1 [file 41398_2024_2939_MOESM22_ESM.pdf]

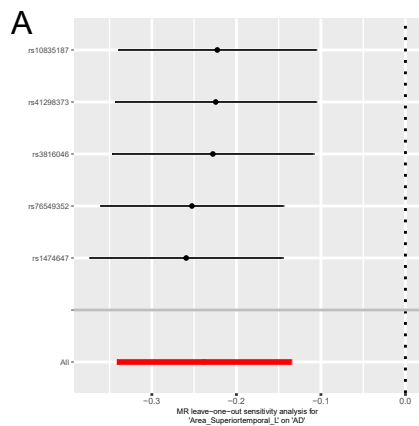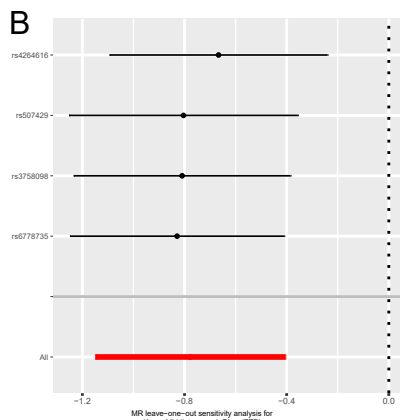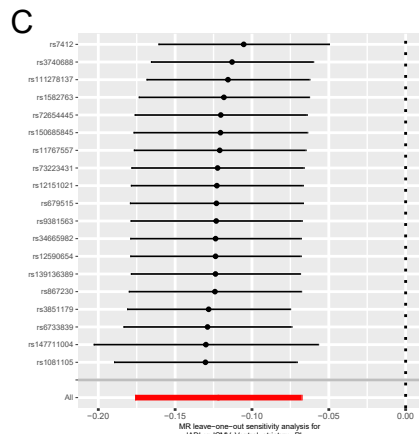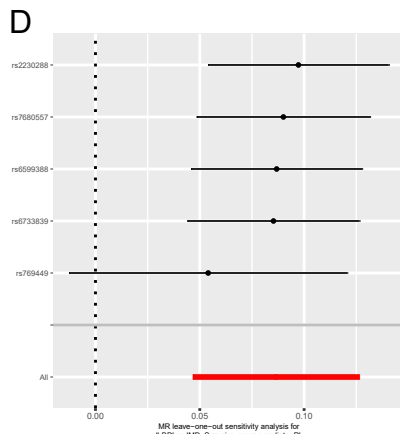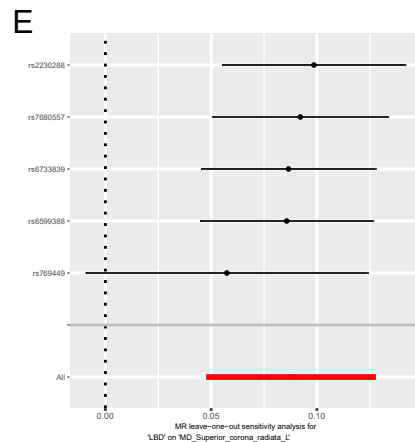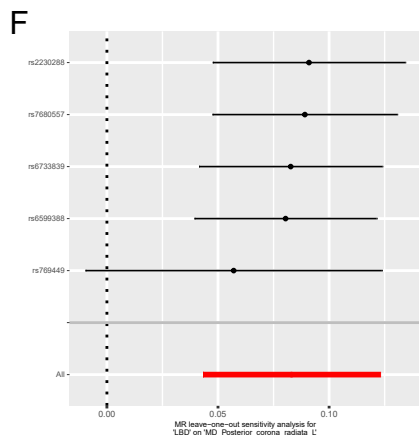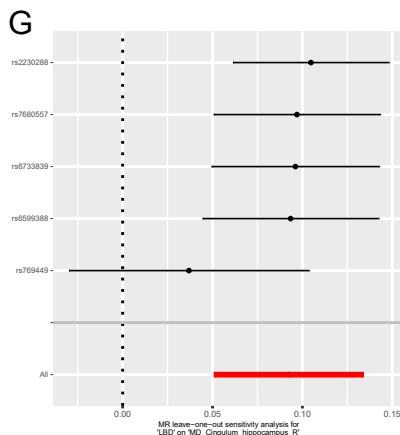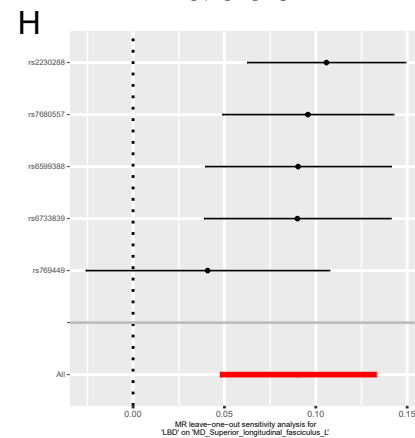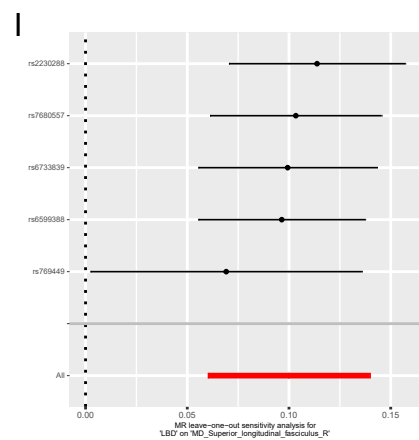

Supplement: Supplementary file 23 — Supplementary Figure 2 [file 41398_2024_2939_MOESM23_ESM.pdf]

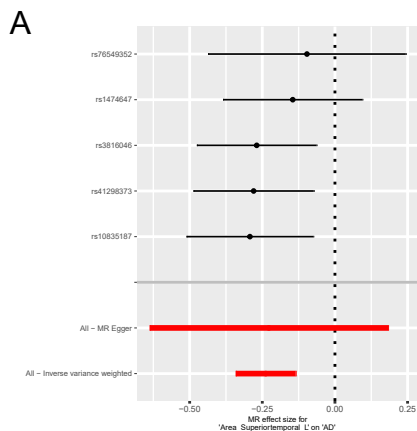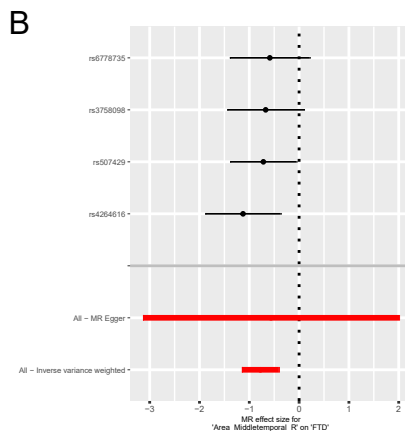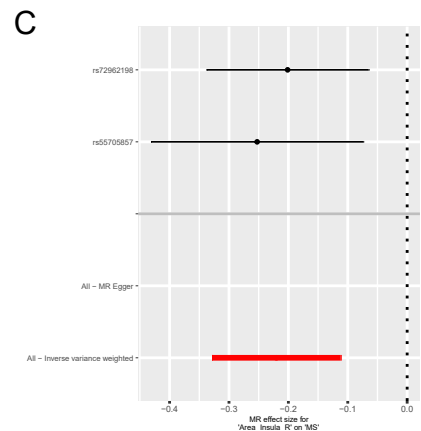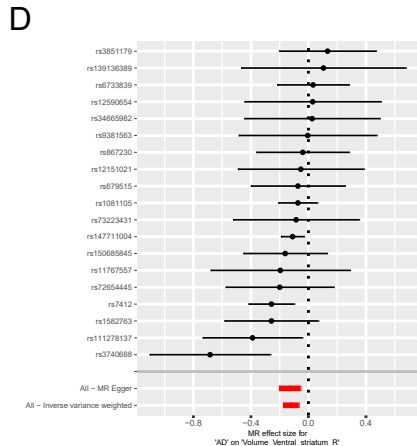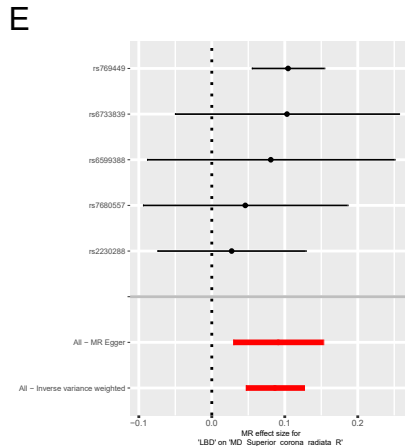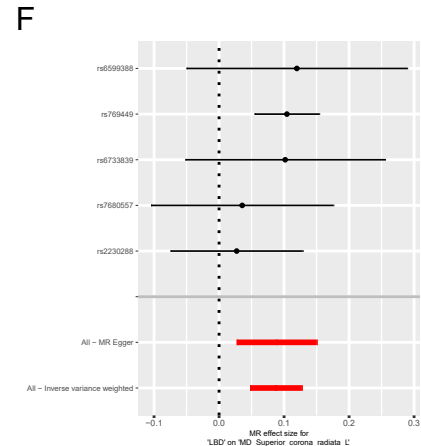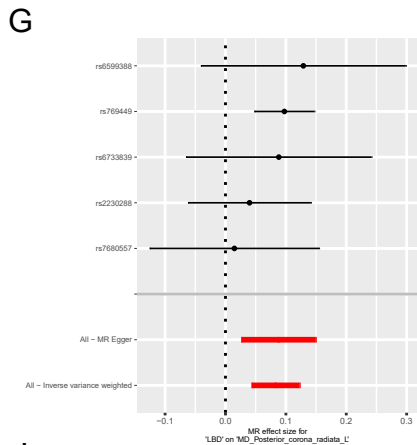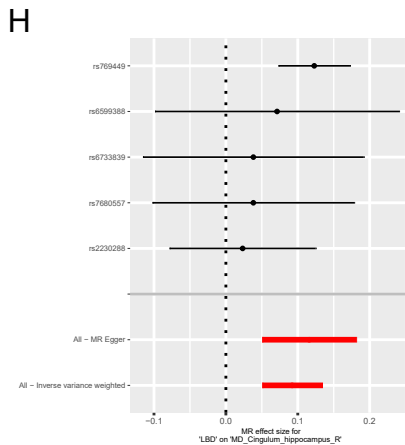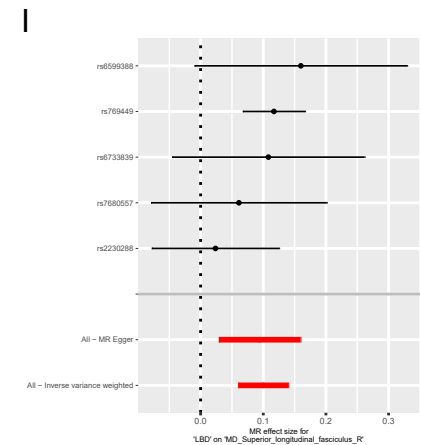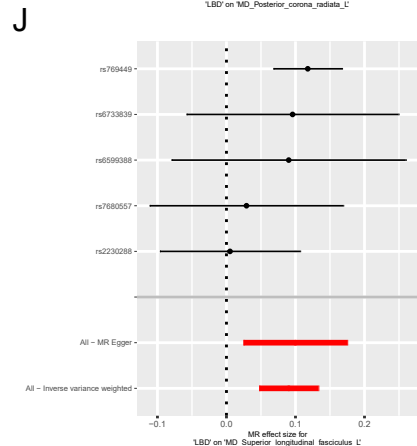

Supplement: Supplementary file 24 — Supplementary Figure 3 [file 41398_2024_2939_MOESM24_ESM.pdf]

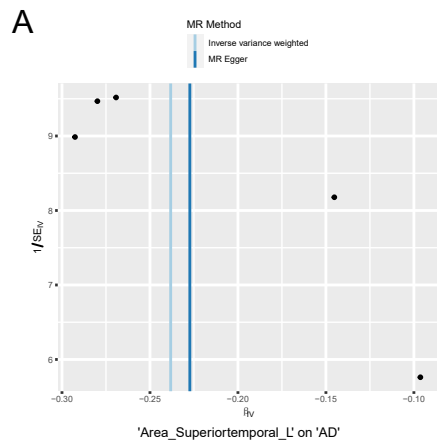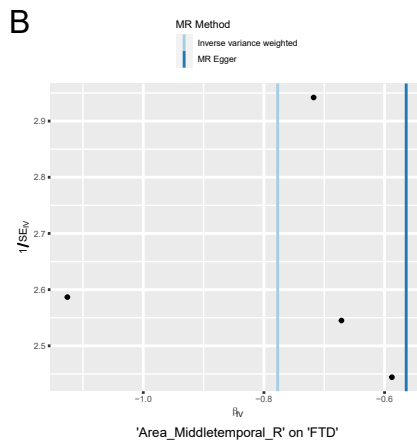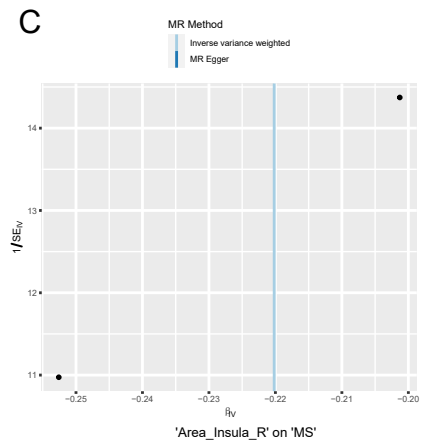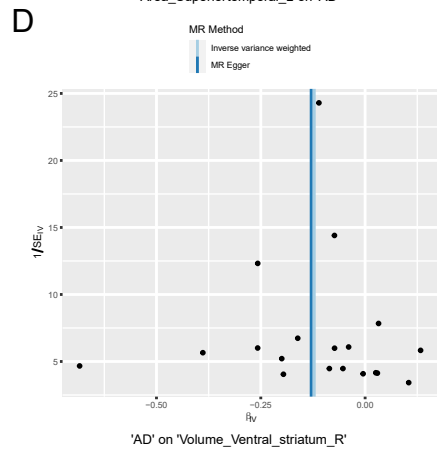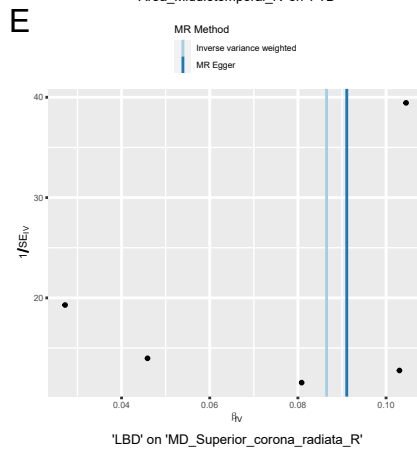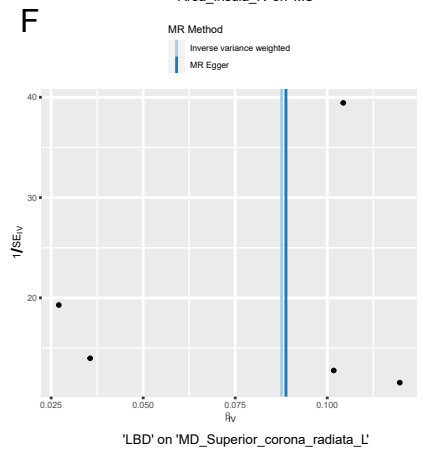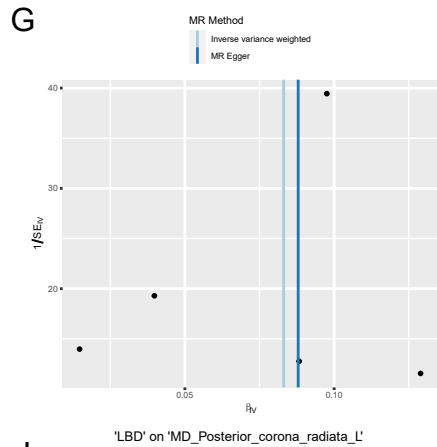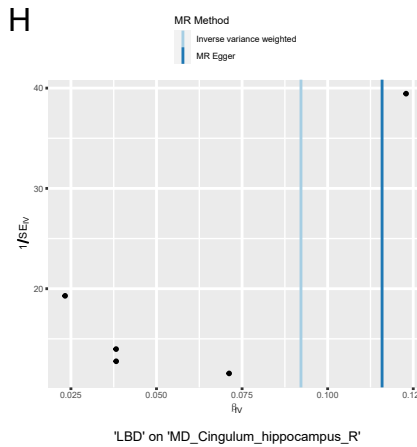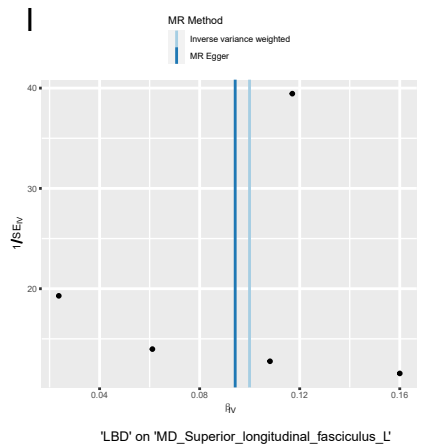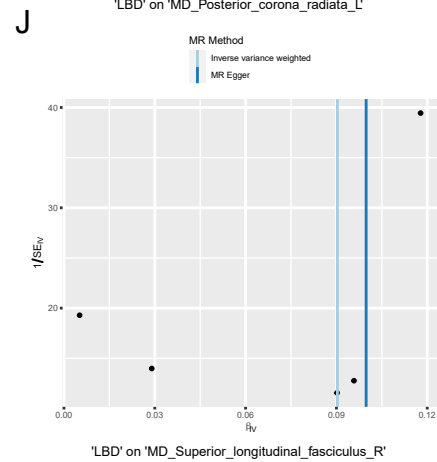

Supplement: Supplementary file 25 — Supplementary Figure 4 [file 41398_2024_2939_MOESM25_ESM.pdf]
